# Supplementary material for: Learning curve and surgical outcome of robotic assisted colorectal surgery with ERAS program
Source: Sci Rep. 2022 Nov 29;12:20566. doi: 10.1038/s41598-022-24665-w (PMC9709162; doi:10.1038/s41598-022-24665-w)
Supplement: Supplementary file 2 — Supplementary Information 2. [file 41598_2022_24665_MOESM2_ESM.zip › RAL Raw data-Ñ[▒K/136/OP_ñw╝╨░O▒Kñσ.pdf]

正式摘要(電子簽章：000000233672812)

W102 / W102

臺中榮民總醫院

Operation Note

[redacted]

- 手術日期：2021/12/29
- 手術醫師：VS 林俊余，CR 林陳彥，CR 陳璿夫，R 蔡濬樟，R 張育豪
- Anesthesia : GE
- Position : Lithotomy
- Aseptic agents : Soap

Diagnosis

- Pre-operative diagnosis : rectum ca s/p SCRT+ FOLFOX
- Post-operative diagnosis : Ditto.s/p robotic LAR+ loop ileostomy

Surgical Indication

- for curative resection

Operation

- 1. Robotic (XI) assisted low anterior resection TME
  2. Lt side pelvic LN dissection
  3. protective ileostomy.

Finding

- 1. A 1 x 1 cm tumor over lower rectal area
  2. There was no obvious visible liver metastasis, no visible peritoneal seeding
  3. No obvious ascites was noted
  4. Intraoperative colonoscopy : (-).; anal retracter was used for check anastomosis and no defect was found, followed with one 3-0 Vicryl interrupt suture upon anastomosis per anus
  4. High ligation (+) : At IMA root. Left colic artery and IMV were also ligated
  5. Take down from the splenic flexure : (-)
  6. Robotic endoGIA (45-4.1) X3 + EEA (29mm) were used
  7. Proximal cut end: 10 cm, distal cut end: 0.5 cm.
  8. ICG perfusion time: distal rectum: 55sec. proximal colon: 45sec
  9. perforation of rectal stump noted, embeded inside the EEA staple circle
  10. Fr. 15 hemovac was inserted from RLQ trocar and placed into pelvic cavity

Procedure

- 1. General endotracheal anesthesia was induced and then the patient was placed in modified Trendelenburg lithotomy position.
  2. Preoperative antibiotics were given. A Foley catheter and nasogastric tube were placed.
  3. The abdomen was prepped and draped in standard sterile fashion.
  4. A small vertical incision was made above the umbilicus and a 8 mm trocar was inserted into the peritoneal cavity by open method. Peumoperitoneum was established with CO2 inflation.
  5. Laparoscopy was inserted and then other two 8mm trocars were inserted over LUQ, RLQ of abdomen. Another 12mm trocars was inserted over RLQ area, and the other assist-port 12mm troca was inserted on RUQ. The operative findings were mentioned as above.
  6. The sigmoid colon was held by grasper and the peritoneum overlying the mesentery was scored with electrocautery.
  7. The inferior mesentery artery was identified, ligated above the left colic artery with endoclips, and transected.
  8. Dissection was done above the Gerota's fascia and toward the peritoneal attachments.

- 9. The colon was mobilized from retroperitoneum overlying the sacral promontory and into the pelvis. The incision was extended laterally and medially across the peritoneal reflection.
10. The mesorectum was then mobilized.
11. Distal descending colon and rectum were mobilized.
12. The mesorectum was then mobilized using sharp dissection until the levator ani muscle was identified.
13. Colonscopy was done for double checking the tumor location.
14. The rectum was divided with Robotic endoGIA (45-4.1) X3.
15. Then do the Lt side pelvic lymph node dissection. loop the Lt side ureter with vessel loop. Dissect alone int. border of ext. iliac artery and vein till obturator muscle. Then protect obturator nerve carfully. Dissection was deepened into pelvic floor, then dissect inner side of pelvic LN Gr 283. Whole lymphnode was smoothly dissected.
16. The midline wound was enlarged and the rectum was pulled out. The proximal colon was then taken out for carefully inspection, and to make sure it has an excellent blood supply. The mesenteric fat was gently cleared from the terminal 2-3 mm of the bowel ends.
17. The field was isolated with moist pad and the bowel opened and sized. It was determined that a 29 mm circular stapler could be accommodated. A purse string suture of 2-0 prolene was placed. The tumor-containing tissue was removed. Then the anvil was inserted, followed by the purse string suture. The colon was then put back to the abdominal cavity.
18. Pneumoperitoneum was constructed again.
19. The anal canal was irrigated with distil water and then the circular stapler was introduced through the anus and pierced through the upper area (The ventral site of the body) of the stapler line.
20. The proximal bowel with anvil in place was engaged with the circular stapler and the stapler was then fired.
21. The desired ileum site (About 15~20cm from IC valve) was selected for further ostomy construction.
22. The 12mm trochar wound over the RLQ area was further extended to creat the site for loop ileostomy.
23. The ileum was retracted from the desired ostomy site. A window was created under the ileum by dividing the mesentery adjacent to the bowel wall with electrical cautery carefully, with a plastic bridge in placed.
24. Fr. 15 hemovac was inserted from RLQ trocar and placed into pelvic cavity
25. Perforation of rectal stump noted, embeded inside the EEA staple circle, The anastomosis was checked under anal retractor and no defect was found, followed with one 3-0 Vicryl interrupt suture upon anastomosis per anus.
26. the fascia of 12mm trochar sites and central extended wound were closed with interrupted sutures via Num. 1 Vicryl.
27. The wound was further closed with 3-O Vicryl, and the skin was closed with 3-0 Nylone.
28. The ileostomy was matured with multiple interrupted mucocutaneous suture via 4-0 Monocryl.
29. The ileostomy was well formed and viable. An ostomy bag was placed
30. The patient tolerated the procedure well and was brought to the POR in stable condition.
- Estimated blood loss: minimum
- 器械數量: 正確
- 紗布數量: 正確

Image

- PAINT
  - abdomen

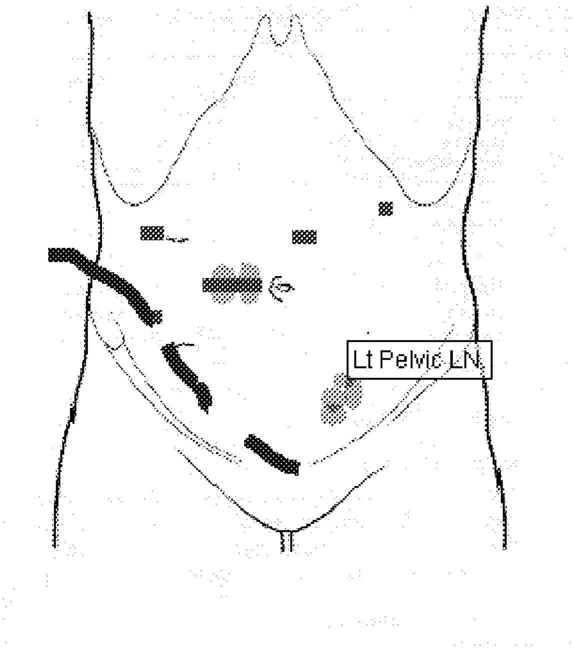

- colon & rectum

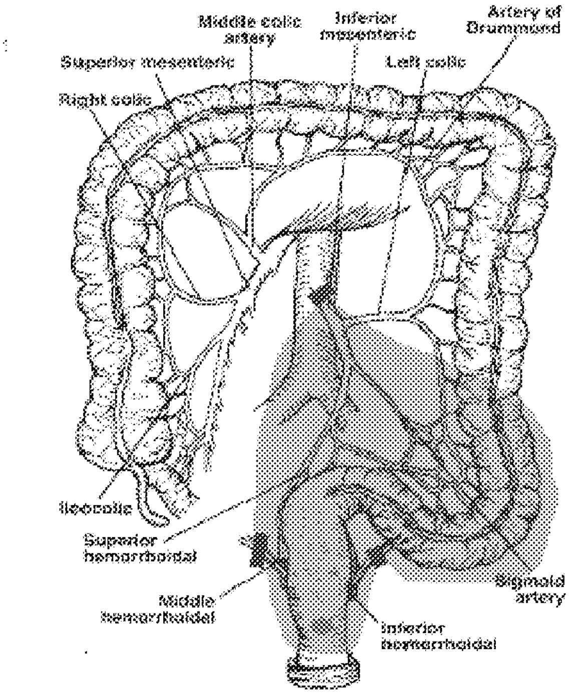

主治 2222H林俊余 / 醫師 2310K陳璿夫

正式摘要(電子簽章：**000000233672812)**

W102 / W102

doctorRank:B
